# Factors associated exclusive breastfeeding practices of urban women in Addis Ababa public health centers, Ethiopia: a cross sectional study

**DOI:** 10.1186/s13006-015-0047-4

**Published:** 2015-07-07

**Authors:** Tigest Shifraw, Amare Worku, Yemane Berhane

**Affiliations:** Addis Continental Institute of Public Health, P.O. Box 26571, 1000 Addis Ababa, Ethiopia

**Keywords:** Exclusive breastfeeding, Addis Ababa, Ethiopia

## Abstract

**Background:**

Exclusive breastfeeding (EBF) is the best nutrition for the children during the first 6 months of life, yet despite knowing the clear benefits, the practice of EBF is low. The aim of the study is to determine prevalence of exclusive breastfeeding practice and associated factors in Addis Ababa.

**Methods:**

A facility based cross-sectional study with internal comparison was conducted among 648 mothers attending immunization sessions in all public health centers in Addis Ababa, Ethiopia, in February 2011. Prevalence of EBF was determined using ‘recall since birth’ method. Multiple logistic regression was used to adjust for confounding effects while determining the association between exclusive breastfeeding practice and selected factors.

**Results:**

The prevalence of EBF under six months was 29.3 % (95 % CI 25.9, 32.9). Mothers whose monthly income 500 - 1000birr (US$56 - 113) were more likely to exclusively breastfeed than those who earn more than 1000birr (US$113) (Adjusted Odds Ratio [AOR] = 2.49; 95 % Confidence Interval [CI] 1.06, 5.88). Mothers who reported having antenatal counseling (AOR = 1.99; 95 % CI 1.16, 3.43) and postnatal counseling were more likely to exclusively breastfeed than those who did not have counseling (AOR = 2.12; 95 % CI 1.28, 3.54). Mothers who gave birth vaginally were more likely to exclusively breastfeed than those who had a Caesarean section (AOR = 2.40; 95 % CI 1.25, 4.61).

**Conclusions:**

The prevalence of exclusive breastfeeding was low in Addis Ababa. Mothers’ income, antenatal and postnatal counseling and mode of delivery were found to be associated with EBF practices. Recommendations include strengthening nutrition counseling during antenatal and postnatal sessions, further exploring the barriers to EBF for higher income mothers and offering continuous assistance and safe pain relief medication for mothers who gave birth by caesarean section.

## Background

Suboptimal breastfeeding practices, especially non-exclusive breastfeeding in the first six months of life, are contributing to the burden of childhood diseases and mortality [1–3] and is responsible for 45 % of neonatal infectious deaths, 30 % of diarrheal deaths and 18 % of acute respiratory deaths [4]. Exclusive breastfeeding can significantly reduce the number of under five deaths in Africa, especially Sub-Saharan Africa where 41 % of deaths occur mainly due to inadequate breastfeeding practices in combination with high levels of disease [5].

World Health Organization recommends that infants start breastfeeding within one hour of life, and be exclusively breastfed for six months, with timely introduction of adequate, safe and appropriate complementary foods while continuing breastfeeding for up to two years of age or beyond [6]. Exclusive breastfeeding means that the infant receives only breast milk. No other liquids or solids are given, with the exception of oral drops of vitamins, minerals or medicines [7].

One of the major causes of malnutrition in Ethiopia is inappropriate child feeding practices and half (52 %) of infants less than six months old were exclusively breastfed [8]. Exclusive breast milk feeding for the first six months of an infant’s life is associated with lower rates of gastrointestinal and respiratory diseases, otitis media and allergies, better visual acuity, speech and cognitive development [9, 10].

Despite its demonstrated benefits, EBF duration in many countries including Ethiopia are lower than the international recommendation of exclusive breastfeeding for the first six months of life, especially in urban areas [11, 12]. Strategies to protect, promote, and support exclusive breastfeeding are needed at the national, health center and community levels [13].

The aim of this study is to determine the prevalence of EBF under six months and the potential factors associated with the practice/duration of EBF among women in Addis Ababa.

## Methods

Facility based cross-sectional study with internal comparison was performed in all public health centers in Addis Ababa, capital city of Ethiopia, in February 2011. The source population is all mothers with children eligible for vaccination in Addis Ababa. The study population included all mothers who attended a public health center for their children’s measles vaccination at nine months of age and the inclusion criteria were mothers who attended a public health center for their children’s measles vaccination during the data collection period.

The sample size was calculated by using a single population proportion formula considering the following assumptions: based on Ethiopian DHS 2011 prevalence of 52 % EBF, with 95 % confidence level and 4 % marginal error. Allowing for a 10 % non-response rate the total planned sample size was 660.

Data were collected using interviewer administered structured questionnaire. The questionnaire was adapted from the child health module of the Ethiopian DHS 2005 and other large-scale National Community Based Nutrition surveys. It assessed the study participants socio-demographic, reproductive and health service characteristics. The questionnaire was prepared in English and translated to Amharic by a panel of public health experts to check its consistency and conceptual equivalence.

Ten nurses were recruited as data collectors and they were trained for three days by the principal investigator on the objective of the study tool and data collection procedures. The interviews were conducted in the compound of a health facility after the mother received the service from immunization room. Each interview took about 15 min on average.

In the Addis Ababa health bureau there are ten sub cities and under these 27 public health centers available. All health centers except one were included in this study and the excluded health center was used for pretesting. The sample was proportionally allocated for each health center based on the number of children came for vaccination previous year.

All mothers who came to each facility for their children’s measles vaccination were invited and included until the required number obtained. The routine delivery of the health service was not disrupted during data collection period.

The principal investigator supervised the data collection regularly. Data were edited and coded manually before entering in to computer software. Double entry was done to reduce error during data entry.

The investigator used “since birth” method to calculate the prevalence of exclusive breastfeeding. The analysis was performed with the SPSS version 15 software package. The prevalence of EBF was calculated by taking mothers whose children were fed only breast milk until six months of age as numerator and all children nine months old whose mothers were interviewed as denominator. Sociodemographic and health service factor were presented with numbers and percentages. Bivariate and multivariate analysis was done to see association between EBF and factors to control for confounders respectively. All variables with *p*-value < 0.2 were entered in the multiple logistic model. The association was presented using crude and adjusted odds ratio with 95 % confidence interval. A *p*-value of < 0.05 was taken to be statistically significant.

Ethical approval was obtained from the University of Gondar. The necessary permission and letter of support to all the study health centers were obtained from Addis Ababa Region Health Bureau. Informed consent was obtained from each study participant after explaining the purpose of the study and assuring confidentiality of their responses. Participating in this research was on a voluntary basis and the risks were minimal.

### Definition of terms

#### Exclusive breastfeeding since birth

An infant fed only breast milk from the mother or expressed breast milk, and not fed on any other liquids or solids with the exception of drops or syrups consisting of vitamins, mineral supplements, or medicines till six months.

#### Predominant breastfeeding

The infant’s predominant source of nourishment has been breast milk. However, the infant may also have received water and water-based drinks (sweetened and flavored water, teas, infusions, etc.), fruit juice, oral rehydration salts solution (ORS), drop and syrup forms of vitamins, minerals and medicines, and ritual foods (in limited quantities). With the exception of fruit and sugar water, no food-based fluid is allowed under this definition.

#### Partial breastfeeding

Giving a baby some breastfeeds, and some artificial feeds, either milk or cereal, or other food.

#### No breast milk

The infant receives no breast milk.

## Results

### Sociodemographic characteristics

Six hundred forty eight mother-child pairs participated in the study with the response rate of 98 %. Mothers mean age was 26.2 years and range of 16 to 40. Ninety three percent of mothers were married, Orthodox Christian 428 (66.0 %), not working 400 (61.7 %), educated at the primary school level 265 (41.0 %) and 456 (73.8 %) had low monthly income (Table 1).

**Table 1:** Sociodemographic characteristics of participants (*n* = 648) in Addis Ababa public health centers, 2011

Variables		Number	Valid %
Marital status	Single	34	5.3
	Married	600	92.7
	Divorced\separated or widowed	13	2.0
Maternal age	15 - 24	244	37.9
	25 - 34	351	54.5
	≥35	49	7.6
Religion	Orthodox Christian	428	66.0
	Protestant	64	9.9
	Catholic	11	1.7
	Muslim	145	22.4
Ethnicity	Oromo	128	19.8
	Amhara	270	41.7
	Tigre	32	4.9
	Gurage/Silte	191	29.5
	Other	27	4.2
Maternal education	Illiterate	151	23.3
	Primary	265	41.0
	Secondary	149	23.0
	Above secondary	82	12.7
Paternal education	Illiterate	58	9.0
	Primary	208	32.2
	Secondary	229	35.4
	Above secondary	151	23.4
Maternal working status	Working	248	38.3
	Not working	400	61.7
Workplace	Home	38	15.6
	Outside home	188	77.4
	Home & outside	17	7.0
Maternal income per month	< 500 birr (US$56)	456	73.8
	500 - 1000 birr (US$56 – 112)	92	14.9
	> 1000 birr (US$112)	70	10.7
Number of living children	1	356	55.0
	2	204	31.5
	≥ 3	87	13.4

### Health service related characteristics

Ninety seven percent received antenatal care during their last pregnancy. Fifty one percent attended a health center for greater than three visits and nurses provided antenatal care for 84 %; 54 % delivered at a health centers.

Ninety eight percent of mothers gave breast milk to their children. The proportion of mothers who gave colostrum to the newborns was 83 % and 14 % of mothers gave expressed breast milk to their children. Eighty percent of the respondents initiated breastfeeding within one hour after delivery, assisted by nurses or midwives. Sixty eight percent received postnatal counseling on child feeding from nurses. Most mothers received advice from a health provider to continue EBF until six months (92 %) (Table 2).

**Table 2:** Health service related characteristics of participants (*n* = 648) in Addis Ababa public health centers, 2011

Variables			Number	Percent
Attended ANC	Yes		630	97.4
	No		17	2.6
Place of ANC	Government hospital		35	5.6
	Health center		535	85.1
	Private health facility		59	9.4
Type of ANC provider	Doctor (626)	Yes	106	16.9
		No	520	83.1
	Nurse/Midwife (625)	Yes	527	84.3
		No	98	15.7
Number of ANC visits	≤3 visits		307	49.2
	>3 visits		317	50.8
Breastfeeding information provided during ANC	Yes		473	73.8
	No		168	26.2
Index child place of birth	At home		55	8.5
	Government hospital		163	25.2
	Health center		350	54.1
	Private hospital/clinic		59	9.1
	Other		20	3.1
Child ever breastfed	Yes		638	98.5
	No		10	1.5
Exclusive breastfeeding since birth	Yes		186	29.3
	No		449	70.7
Colostrum given	Yes		536	82.7
	No		112	17.3
Breastfeeding initiation	<1 hour		483	80.6
	1 - 2 hours		40	6.7
	2 - 23 hours		59	9.8
	>24 hours		17	2.8
Child ever been given expressed breast milk	Yes		92	14.2
	No		555	85.8
Postnatal follow-up	Yes		416	64.2
	No		232	35.8
Postnatal counseling on child feeding received	Yes		440	68.2
	No		205	31.8

*ANC* Antenatal clinic

### Prevalence of exclusive breastfeeding practice at six months

The prevalence of exclusive breastfeeding since birth was 186/635 (29.3 %) (95 % CI 25.9, 32.9), predominant breastfeeding 44.3 %, partial breastfeeding 24.9 % and no breastfeeding 1.5 %.

In multivariate analysis, mothers’ income, recieving antenatal and postnatal counseling about infant feeding, and mode of delivery were independent factors associated with exclusive breastfeeding practice. Other sociodemographic factors including maternal age, maternal education, father’s income and number of children were not significantly associated with six month of exclusive breastfeeding practice.

Mothers with mid-range income were 2.5 times more likely to exclusively breastfeed than mothers with higher (AOR = 2.49; 95 % CI 1.06, 5.88). Mothers who received antenatal counseling about breastfeeding were 2 times more likely to exclusively breastfeed compared to those who did not have counseling about infant feeding during ANC (AOR = 1.99; 95 % CI 1.16, 3.43). Mothers who gave birth vaginally were 2.4 times more likely to exclusively breastfeed than those mothers who had C-section (AOR = 2.40; 95 % CI 1.25, 4.61). Mothers who had postnatal counseling about infant feeding were 2.12 times more likely to exclusively breastfeed compared to those who didn’t have counseling about infant feeding during postnatal care (AOR = 2.12; 95 % CI 1.28, 3.54) (Table 3).

**Table 3:** Factors associated with exclusive breastfeeding practice among participants (*n* = 648) in Addis Ababa public health centers, 2011

Variables		EBF (Yes)	EBF (No)	Crude Odds Ratio (95 % CI)	Adjusted Odds Ratio (95 % CI)
Maternal occupation	Working	45	144	1.00	
	Not working	133	276	0.67 (0.46, 0.96)	
Maternal income	Low	130	319	2.41 (1.13, 5.19)*	1.97 (0.91,4.31)
	Middle	29	58	1.97 (1.02, 3.78)*	2.49 (1.06, 5.88)*
	High	12	58	1.00	1.00
Place of work	Home	40	141	0.59 (0.39, 0.89)*	
	Outside home/both	146	307	1.00	
Antenatal counseling about breastfeeding	Yes	156	307	2.50 (1.59, 3.93)**	1.99 (1.16, 3.43)*
	No	28	138	1.00	1.00
Mode of delivery	Vaginal	158	321	2.11 (1.21, 3.64)**	2.40 (1.25, 4.61)**
	C - section	18	77	1.00	1.00
Place of birth	Health center	121	224	3.00 (1.42, 6.3)**	
	Gov. hospital	41	120	1.89 (0.86,4.19)	
	Private health facility	15	51	1.63 (0.66,4.08)	
	Home	9	50	1.00	
Postnatal counseling about infant feeding	Yes	154	278	2.91 (1.89, 4.45)**	2.12 (1.28, 3.54)**
	No	32	168	1.00	1.00

Variables adjusted for in multivariate analysis: maternal age, maternal occupation, maternal income, number of children, place of work, place of ANC, ANC counseling about breastfeeding, mode of delivery, place of birth, postnatal counseling about infant feeding

*p < 0.05, **p < 0.01

## Discussion

Exclusive breastfeeding for the first 6 months of life is the optimal way to feed infants and there are many benefits of breastfeeding [6]. In this study, we found the prevalence of exclusive breastfeeding practice was 29.3 %. In multivariate analysis, maternal income, antenatal counseling about breastfeeding, mode of delivery and postnatal counseling about infant feeding were significantly associated with the EBF practice.

The 29 % prevalence of exclusive breastfeeding at six months of age was lower than that found in the Bahir Dar study [14]. The prevalence of predominant breastfeeding was 44.3 % which is my result finding not Bahir Dar study and it's equivalent to Ethiopian Demographic Health survey 2011 EBF national figure [8]. The DHS calculated exclusive breastfeeding over a 24 h recall period but this study, as in the Bahir Dar study, used recall since birth. Recall since birth is consistent with the World Health Definition of EBF and is useful to link infant feeding patterns with vertical transmission of diseases or latter development of infant allergy. On the other hand, the reporting of the previous 24 h appears to exaggerate the prevalence of exclusive breastfeeding at six months [15].

Maternal income was significantly associated with EBF practice in this study. In other studies there was no relation of EBF with the income of mother [14]. This study indicates that when the mother earned a high income, the rate of exclusive breastfeeding decreased. A possible explanation for this finding is that women with higher income are less likely to stay home during the day time and that may compromise the practice of exclusive breastfeeding. Another factor may be that infant formula and cow milk are not affordable unless the family have higher income.

Exclusive breastfeeding practice was associated with breastfeeding counseling during antenatal visits and postnatal visits, but not with the number of ANC visits. Similar findings were observed in Bahir Dar, Debre Markos northwest Ethiopia, India and Tanzania. Mothers informed about breastfeeding at the antenatal clinic had better feeding practices. Residing in an urban area might be a proxy measure of receiving greater exposure to information from different media channels or greater utilization of health services with staff better trained on infant feeding [14, 16, 17]. This shows that counseling at the facility is important to improve maternal knowledge and breastfeeding practices.

This study revealed that mothers who gave birth vaginally were more likely to practice EBF than those who gave birth by cesarean section. This finding was consistent with a study from Bahir Dar [14]. This could be due to the pain the mother experiences and may delay giving of breast milk and start other formula or cow milk in the first few days to the baby.

We might have introduced recall bias as we sought information about breastfeeding from the mothers since the time of birth. The finding of this study may under estimate the prevalence of EBF practice as the method included all infants as no longer exclusively breastfed as soon as anything else is fed to them. We can generalize the findings for Addis Ababa and other similar settings as the study respondents were drawn from all health centers of Addis Ababa.

## Conclusion

The prevalence of exclusive breastfeeding practice is low. Having antenatal and postnatal counseling about breastfeeding, mother income and mode of delivery were found to be associated with better exclusive breastfeeding practice. Therefore, we suggest strengthening the nutrition counseling during antenatal and postnatal sessions, offering continuous assistance and safe pain relief medication for mothers who gave birth by caesarean section and exploring further the barriers to EBF for higher income mothers.

## Abbreviations

AOR: Adjusted odds ratio
CI: Confidence interval
DHS: Demographic and health survey
EBF: Exclusive breastfeeding
WHO: World Health Organization
